# Smart packaging materials based nanoencapsulated bromothymol as monitoring sensors for spoilage of chilled fillet

**DOI:** 10.1007/s13197-025-06339-3

**Published:** 2025-06-25

**Authors:** Saber Ibrahim, Hager Fahmy, Khaled Elkhawas, Ahmad Labeeb

**Affiliations:** 1 Packaging Materials Department, National Research Centre, Elbehouth Street 33, Dokki, Cairo 12622 Egypt; 2 Nanomaterials Investigation Lab., Central Laboratory Network, National Research Centre, Dokki, Giza, 12622 Egypt; 3 Department of Advertising, Printing and Publishing, Faculty of Applied Arts, Benha University, Banha, Qalubia 13518 Egypt; 4 Animal Health Research Institute, Dokki, Cairo Egypt; 5 Microwave Physics and Dielectrics Department, National Research Centre, Elbehouth Street 33, Dokki, Cairo 12622 Egypt

**Keywords:** Smart packaging, Nano-encapsulation, Food sensory, Intelligent label, Spoilage

## Abstract

Food preservatives have long been used to prevent spoilage due to microbial activity. Detecting spoilage without opening packaging is increasingly important. Smart packaging, a third-generation innovation, was developed to improve spoilage detection. A smart label was created using encapsulated polymer/bromothymol blue and analyzed using Attenuated Fourier Transform Infrared (ATR-FTIR) spectroscopy. In acidic and basic media, significant shifts in the -OH group on the benzene ring were observed (840 to 813 cm^− 1^). Ultraviolet-visible (UV-Vis) spectroscopy showed shifts in maximum absorption at 610 and 411 nm, depending on pH. The label’s topography was studied with a polarized optical microscope (POM), and dynamic light scattering (DLS) revealed nano-encapsulation particle sizes of 63–84 nm with a unimodal distribution. Additionally, a strong positive correlation (*P* < 0.5) was found between aerobic plate count and pH (0.82), demonstrating a connection between microbial activity and food quality. Bromothymol blue capsules (BC) effectively visualized pH changes, confirming their potential as indicators for food spoilage. Sensory evaluation of BC indicates its suitability as a safe and effective intelligent label for monitoring chilled chicken fillets.

## Introduction

Chilled poultry products is one of the highly unpreserved foods, which its shelf life usually depends on factors related to raw materials, handling, processing and storage conditions (Piñon et al. 2018). The shelf life of fresh chicken stored at 4 ^o^C is barely 6 days (Zhang et al. 2022; Rukchon et al. 2014). However, during storage, the spoilage microorganisms proliferate quickly and generate the metabolites that give food its unpleasant smells and scents, which lead to sensuous rejection (Huang et al. 2019). The concentration of the metabolites causes spoilage and can be used as an objective of the chemical spoilage index (Ambrosio et al. 2022). Fast response for any change in the safety and quality of food due to change of pH based on nano and micro capsulations of pH indicators is required. These nano or micro capsulations are impeded into the packaging itself. Even when food appears to be in good condition, this technique detects the early stages of spoiling.

Proteolytic activity of spoilage microorganisms degrades proteins, which suffer further oxidative deamination, and desulfurization, resulting in gases such as NH_3_, and H_2_S. These odorous gases are a sign that meat is starting to spoil (Ibrahim et al. 2021; Alfei et al. 2020). The total volatile basic nitrogen (TVB-N) parameter is one of the most widely used in food quality evaluation The nitrogenous compounds are still the best chemical indicators that are relatively simple to be analyzed and could be employed as sensing of the microbial growth in fresh chicken under aerobic conditions (Poyatos-Racionero et al. 2018; Ibrahim and El-Khawas 2019a, b).

The increase in pH of media due to presence of microorganisms and their metabolites is considered as a good indicator for spoilage under aerobic storage (Yang et al. 2023; Mansur et al. 2019; Zhang et al. 2023). Foods that include free amino acids and enhanced proteins, like chicken lean, frequently exhibit an increase in acidic pH when the number of spoiling microbes rises because of their proteolytic activity (Christensen et al. 2022).

The idea of pH dyes changing color can be used as a detector for volatile acids and bases (Abdellatif et al. 2020). These dyes (ex. bromothymol blue) display an irreversible change in visual appearance (Rukchon et al. 2014; Nopwinyuwong et al. 2010). visual monitoring freshness throughout the storage period, this spoilage indicator gives food goods an efficient shelf life. Consumers, producers, and retailers prioritize food product safety guarantees to preserve their brand value and avoid customer displeasure.

Polymer encapsulation is one of new trends in pH indicators for smart packaging materials applications (Ibrahim et al. 2019, 2021). There are many types of polymer encapsulation based on ecofriendly (aqueous medium) using emulsion polymerization (Ibrahim et al. 2018). Thus, a successful emulsification will yield a prospected homogeneity distribution of encapsulated materials in polymer matrices (Goh et al. 2012). More well-shape micelles formation, more well-defined nanocapsules would be produced after polymerization reaction. Therefore, pH indicator sensitivity and its properties would be enhanced (Ibrahim and Soliman 2016).

Due to its simplicity, affordability, and effectiveness which may be seen with the naked eye without the need for complex detectors, colorimetric detection has gained popularity in recent decades (Mallick et al. 2022). For that, there are highly requirements to established color change indicator for global detection in different fields. On the other hand, clearly deficient in smart colorimetric research area specially in smart packaging application (Ibrahim et al. 2021; Ibrahim and El-Khawas 2019a, b).

In this paper, merging the smart packaging sensory of food packaging and polymer technology for making nanoscale pH indicator into studying the development spoilage processes. These kind of pH indicators are based on of the color variations visually for the food products packaging applications. Thus, this work was scanning the encapsulation processes of bromothymol as pH indicator label laid on stretching polyethylene packaging films. The indicator was encapsulated in PBA throughout nano-emulsion polymerization reaction. The pH label that was created using microbiological analysis of the packaging of chilled chicken lean and color changes were regarded as indicators of spoilage. The surface morphology of the indicators-polymer capsules was studied using scanning electron microscope and polarized optical microscope.

## Materials and methods

### Materials

Butyl acrylate (Sigma, Germany) was distilled over CaH_2_ before used. Bromothymol blue, sodium dodecyl sulfate (SDS) and 2,2′-Azobis(2-methylpropionitrile) (Sigma, Germany), ethanol, methanol, and chloroform were used without further purification. Chicken breast fillet without skin as chicken lean were obtained from local manufacture and stored under refrigeration.

### Preparation of smart packaging film

Encapsulation reaction was done according to (Soliman and Ibrahim 2016) with little modification. BC was dissolved in ethanol with different concentrations (0.01, 0.03 and 0.05 mol. L^− 1^). 20 ml from bromothymol blue solutions were mixed with 80 ml Millipore water 18MΩ containing (0.037 mol) SDS and (0.21 mol) butyl acrylate monomer at 75 °C. The initiator 2.1 × 10^− 2^ mol was dissolved in 0.7 ml Millipore water and added drop wise over 15 min and continued for 6 h under reflux. After that, the reaction was immersed in ice bath and encapsulated bromothymol (EBC) was precipitated in methanol. The precipitate was dried under vacuum at 50 °C.

The prepared EBC was dissolved in chloroform (20 wt %) for 30 min over magnetic stirrer. Then, polybutylacrylate (PBA)/pH indicator solution was purged with nitrogen and sonicated for 10 min. The solution was casting in Teflon Petri dish and dried in vacuum oven overnight. Small round film pieces were used as indicator pads. All pads were attached to stretching polyethylene film by pressing under mechanical hydrolytic piston at room temperature for 10 min.

The design of packaging label was constructed in the Adobe Illustrator CC 2018 program in cyan, magenta, yellow and black (CMYK) colors with spot colors. The label package was printed on flexographic machine Mira flex 2014, Germany. The surface printing was applied on paper ARROW 1114 (GLOSSY). The rate of color change ∆E was measured with X-Rite exact, Pantone, USA for the packaging label.

### Characterization of smart packaging film

The pH indictor EBC composite films in both acidic and basic media were investigated by Bruker FT-IR spectrometer model Vertex-70 instrument in region range of wave numbers 400–4000 cm^− 1^. The main advantage of ATR is the fact it does not demand a deep penetration (0.5 μm to 3 μm) (Badillo-Sanchez et al. 2019). These crystals have very high relative refractive index from 2.38 to 4.01 at certain angle$\:{\:\theta\:}_{c}$related to the sample.

1.1 $$\:\:\theta\:c={\mathrm{sin}}^{-1}\left(\raisebox{1ex}{${n}_{2}$}\!\left/\:\!\raisebox{-1ex}{${n}_{1}$}\right.\right)$$

Where n_2_ is refractive index of the sample, n_1_ is refractive index of the crystal and$\:\:{\theta\:}_{c}$ is critical angle. Optical absorption spectra of PBA/BC films were recorded in range 200–1000 nm using JASCO 630 V UV-Vis-NIR spectrophotometer and Leica DM750P polarizing optical microscope (Leica Microsystem GmbH, Switzerland). In addition, the color change was measured with X-Rite exact, Pantone, USA for two colors. The morphology and topography of EBCs were analyzed by scanning electron microscopy (SEM, Quanta FEG 250, FEI).

### Overall migration

Many international regulations have been legalized regarding the migration of specific substances such as heavy metals, degradation products and additives from the packaging material to the contents of the package. These materials could have bad taste, odors, or suspected harmful effects to consumers. The overall migration (OM) simulants and conditions as detailed in EU Regulation Nr. 10/2011 (The PIM), Simulant A, Simulant B, Simulant D2. All samples were compared to blank sample (Millipore water with resistivity 18.5 MΩ) as reference. The overall migration is expressed as the amount in milligrams of material lost from one decimeter square surface (mg/dm^2^). Overall migration results were calculated according to (EN 1186-5-single side contact in cell test).

### Spoilage examination of packaged chilled chicken fillet

Samples of chicken fillets were made by obtaining fresh, skinless fillets from nearby producers and bringing them to the lab within two hours while refrigerated at test over 6 days storing. When the chicken fillet’s pH was checked when it arrived, the average was 5.82. After that, it was split up into groups and packed right away. A qualitative five trained test panel evaluation of the samples was done for the color, odor, and texture features, then the average was recorded as overall sensory score ranging from 5 = very good, 4 = good, 3 = accepted, 2 = dislike to 1 = very dislike.

Total volatile basic nitrogen (TVB-N) was determined according to European regulation. The pH was determined according to association of official analytical chemists (AOAC). APC was done according to the American Public Health Association (APHA) using peptone water (Oxoid) (1:10) and pour plate technique onto plate count agar (Oxoid) and incubating at 35^o^ C for 48 h (Badillo-Sanchez et al. 2019).

### Label design for packaged chilled chicken fillet

The design of packaging label was constructed in the Adobe Illustrator CC 2018 program in cyan, magenta, Yellow and black (CMYK) colors with spot colors. The label package was printed on flexographic machine Mira flex 2014, Germany. The surface printing was applied on paper ARROW 1114 (GLOSSY). The rate of color change ∆E was measured with X-Rite exact, Pantone, USA for the packaging label.

## Results and discussion

### ATR-FTIR spectrometry

**Fig. 1:**
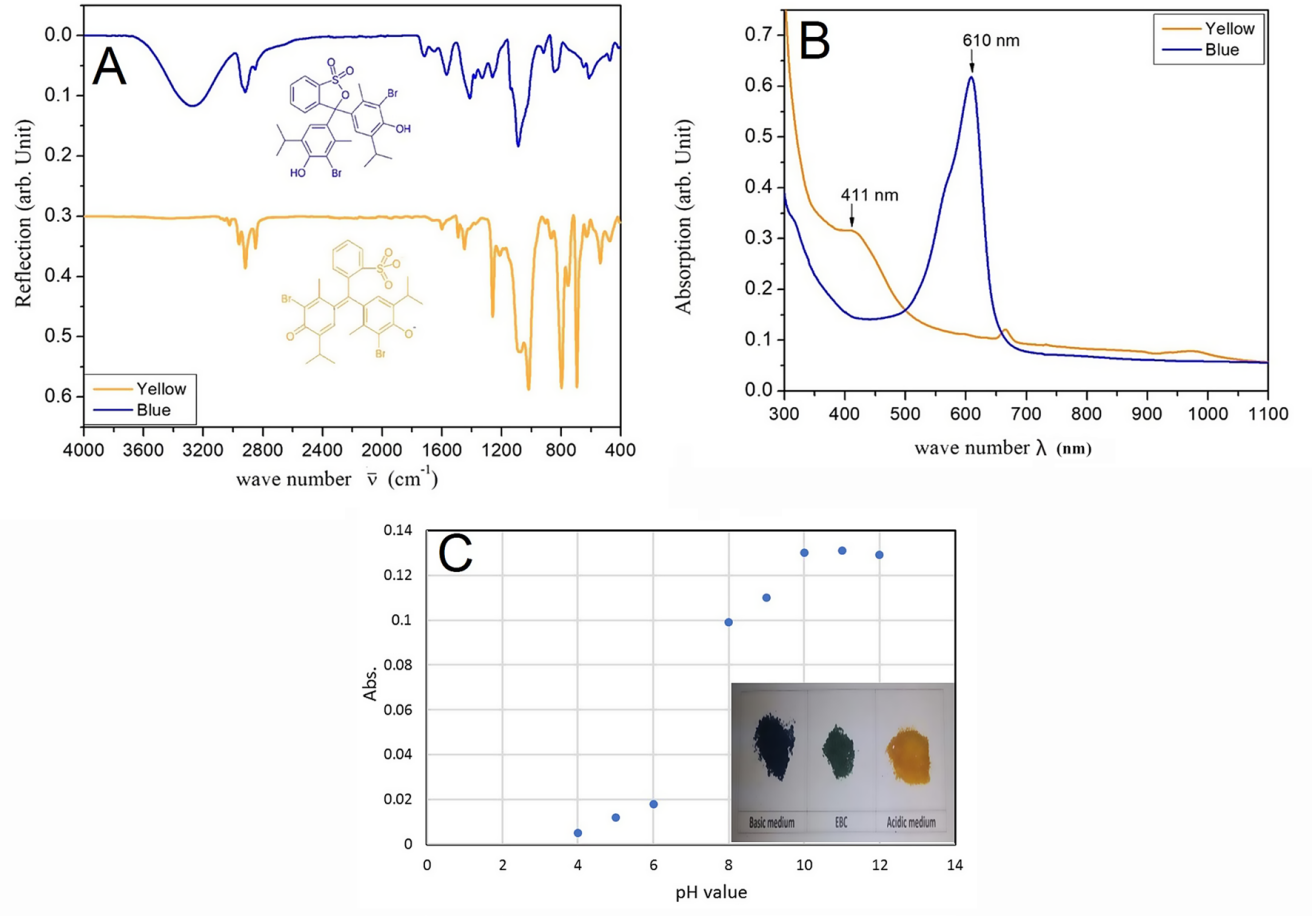
**A**) The vibrational spectra of bromothymol blue encapsulated in poly butylacrylate in both acidic and basic media. The blue curve shows the spectrum in basic medium and related structure, while yellow spectrum represented acidic medium and related structure. **B**) The UV-VIS spectra of bromothymol blue encapsulated in the poly butyl Acrylate as pH indicator for acidic media (yellow line) and as pH indicator of basic media (blue line) for food packaging Validity sensors. **C**) Encapsulated bromothymol blue in different pH mediums

The vibrational spectra of EBC in PBA matrix for both acidic and basic media were measured to examine the functional and chemical structure as influences of pH variations. In Fig. (1a) at frequency range 3100–3600 cm^− 1^ a strong broad peak of hydroxyl group as phenol/alcohol feature stretching vibrational peak to be appeared at 3265 cm^− 1^ as indication of intermolecular hydrogen bonding (Nyquist 2001). While it disappeared in acidic media which carbonyl (-C = O) weak stretching peak located at 1600 cm^− 1^. Moreover, there is a shift of carbonyl groups to be at 1567 cm^− 1^ in basic as strong peak media rather than 1600 cm^− 1^ in acidic media as weak peak. Both C-H stretching peak modes observed at 2840–3022 cm^− 1^ with their overtone as featuring aliphatic and aromatic types. Whereas the C-H bend vibration is observed at 840 cm^− 1^ in basic medium but shifted to 813 cm^− 1^ in acidic medium due to reversible change in chemical structure of the pH indicator in polymer network. On the other hand, S = O stretching mode is detected at 1264 cm^− 1^ as strong vibration in acidic medium and its overtone is in fingerprint region also at 1029 cm^− 1^ as overlapped to be a strong spark with C-O Strong stretching vibrational mode. However, in the basic medium the S = O is observed to be stretching peak at 1264 cm^− 1^ as sulfoxides and its overtone stretch peak to be as shoulder within C-O vibration mode at 1029 cm^− 1^ sulfones(Hasegawa 2017). Although S = O appears in both acidic and basic but regarding to the bromothymol blue structure in different pH, the basic medium was too weak if it is compared with acidic. This evidence of variation in color with change in pH indicator can be attributed to chemical structure that affect by acidic or basic medium even encapsulated form of pH indicator in polymer matrices(Heck and dos Santos 2023).

### UV-Vis absorption

As shown in Fig. 1b, absorption peak of the indicator in the basic medium is located at 411 nm. On the other hand, in the acidic medium, the EBC in the PBA as pH indicator gives pale yellow absorption peak at 610 nm. The isosbestic point was shown at 499 nm which indicated to the bromothymol blue alone (Kodeh and El-Nahhal 2019). The presence of polymer matrix as confinement environmental for bromothymol blue leads to a slightly shifts in absorption peaks within small changes in the pH medium.

### Color change investigation

The free bromothymol blue indicator exhibits a yellow color in acid solution and blue color in base solution (Singh et al. 2011; Moloney et al. 2018). As shown in Fig. 1c, the absorbance of encapsulated indicator gave the highest response at pH 10–12, where the absorbance decreased below or above pH range 10–12. With a high degree of accuracy, the colorimetric sensor for color change distinguishes between blue and yellow very easily. This nice and comfortable eye differentiation between two colors can be attributed to position of in CIELAB chart (Hager 2021). Yellow (L = 52.92, a = 8.85, b = 54.53) and blue (L = 38.71, a = 10.11, b=-32.24) are represented on the b* axis as þb* (positive) and Àb* (negative) values, respectively. Results of pad color was evaluated trough comparison with Media standard print 2020 CIE L, a, b color values of the solid base color for sheeted, web and continuous grades (ISO 12647, 2020). The rate of color change (∆E) between measured colors samples and standards making the measurements within the permissible limits according to ISO 12,647. The results shown that, the colors are (cyan ∆1.79), and (yellow ∆4.16). This is given easy and comfortable eyes distinguish between blue and yellow color.

### Polarized optical microscopy

**Fig. 2:**
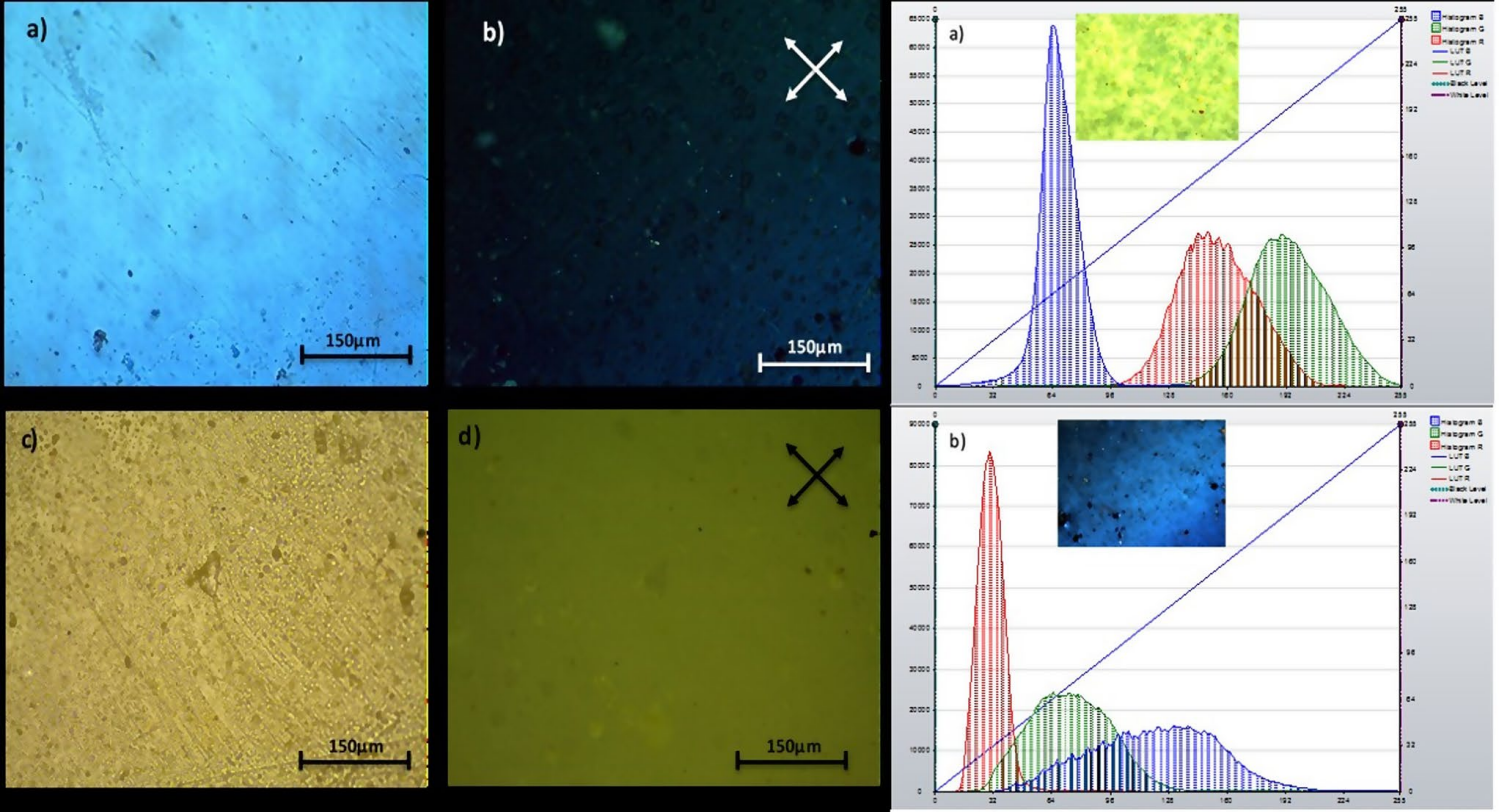
The polarizing optical microscope of bromothymol blue encapsulated poly butyl acrylate in basic medium, **a**) without crossed polarizers, **b**) under crossed polarizers. On the acidic medium yellow colored bromothymol blue in poly butyl acrylate, **c**) without crossed polarizers, **d**) under crossed polarizers and the color histogram for basic light colors red, green and blue RGB for **a**) polymeric nano-capsules indicator in acidic media and, **b**) polymeric nano-capsules indicator in basic media

PBA is isotropic material so that it is dark in between crossed polarizers (Prokhorov et al. 2018), the presence of bromothymol blue which is anisotropic materials so that can manipulate the light direction under crossed polarizers (Kodeh and El-Nahhal 2019). In Fig. 2, the POM photos were taken and measured all in ambient temperature at 20 ^o^C. Figures 2a) and 2b) are representing the encapsulated bromothymol blue in polymer matrix that is dispersed uniformly in the basic medium. There are some powder clusters of pH indicator which are dispersed as bright dots and appear under crossed polarizers only as shown in Fig. 2b whereas barely seen in Fig. 2d. Figure 2c) and 2d). The true colors as blue and yellow of polymer composites are observed in the 4a and 4c when no polarizers are applied. The RGB histogram consists of distribution of each primary color’s brightness level in the image (RGB or red, green, and blue).

In Fig. 2, the acidic media formation is determined by yellow color which the RGB histogram analysis provided green and red colors combination to show yellow color with low blue ratio. This mean the majority color is pale greenish yellow as in Fig. 2a. On other hand, in the basic media the majority color is blue with few green color contributions which has the highest RGB range in active band ratio under the diagonal line between black level and white level in the histogram analysis as shown in Fig. 2b.

### Particle size of nanocapsules

Dynamic light scattering (DLS) is highly trusted technique for investigation the actual particle size. On other hand, microscopic techniques TEM and SEM for nanomaterials materials detection have a little conviction as a selected image result. For that, DLS has deep certainty to give a complete and tangible profile of particle size.

**Fig. 3:**
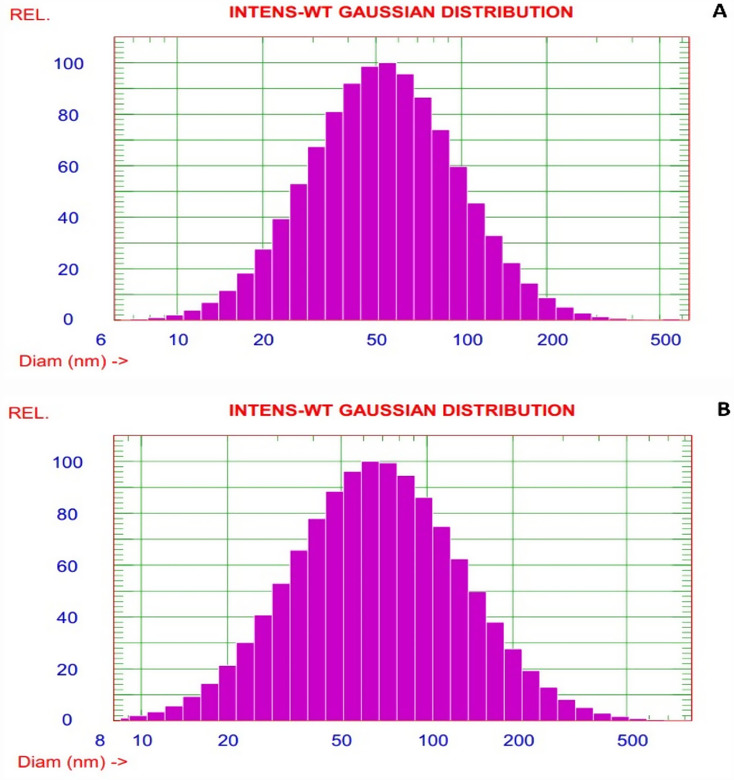
Particle size distribution of (**A**) polybutylacrylate PBA and (**B**) encapsulated bromothymol EBT

Figure 3. the particle sizes were measured by DLS, and the results presented in range 63.5 nm and 84.5 nm for PBA and EBT respectively as nanocapsule indicator. In addition, excellent homogenized and narrow distributions as unimodal bell curve (Meawad and Ibrahim 2019) are indicated with low PDI 0.365 and 0.468 for PBA and EBT respectively.

### Scanning electron microscope

Direct microscopic observation of BCs encapsulated in polymer composites is not easy due to the intense differences in behavior and particle dimensions of the BCs. Topographic based methods, such as scanning electron microscopy (SEM) only show a cross section of the nearly one-dimensional BCs film. These problems make it difficult to observe an entire BCs or to distinguish between different BCs and difficult to conclude from micrographs.

**Fig. 4:**
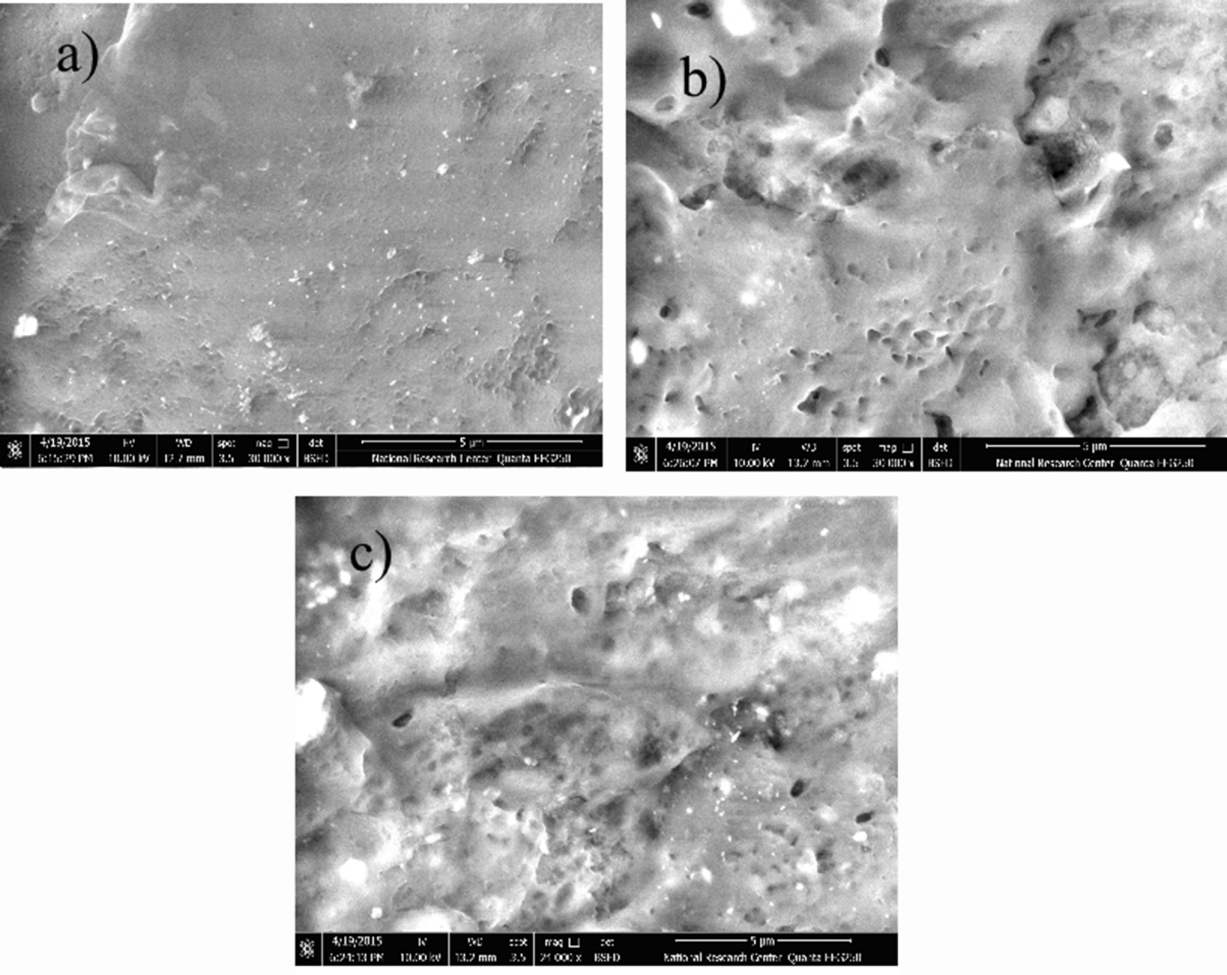
Shows the topographic micrograph of encapsulated BC with different ratios (**a**) BC1, (**b**) BC2 and (**c**) BC3

The topographic images of morphological structure for EBT with different ratios was presented in Fig. 4. The smoothness of the film surfaces was varied from nice flat homogeneous film in bromothymol blue capsule BC1 to more heterogeneous film with high indicator in BC2 and BC3. Encapsulation of organic and or inorganic materials had direct effect in the surface film performance (Ibrahim and Soliman 2016). The holes and caves in the morphological image can be indicated to nucleation of nanocapsules which trapped solvent through drying process.

### OM test of coated paperboard

One of a vital character for any prospective packaging materials is evolution the migrated substance from packaging film to packaged items. For that, the overall migration test was applied to prepared smart films according to EC 10/2011. The migrated values of substances from bulk films to one-side contact simulants are presented in Table 1. The water simulant was extracted with 20% ethanol at concentrations ranging from 1.03 to 2.18 mg/dm^2^. All determined migrated values were under the acceptable limit according to the EC 10/2011 standard. These results, including smart color change area, give a strong recommendation to apply prepared smart packaging materials in food-contact applications.

**Table 1:** The overall migration from smart packaging

Method Replicates	EN-1186-5 Migration into 10% v/v ethanol (simulant A) mg/dm^2^	EN-1186-5 Migration into 20% v/v ethanol (simulant A) mg/dm^2^	EN-1186-5 Migration into 3% w/v acetic acid (simulant B) mg/dm^2^	EN-1186-4 Migration into Olive oil (simulant D_2_) mg/dm^2^
1	2.8	3.4	4.2	0.1
2	2.9	3.5	4.3	0.1
3	2.7	3.6	4.1	0.1
Mean result	**2.8**	**3.5**	**4.2**	**0.1**
Limit	**10.0**	**10.0**	**10.0**	**10.0**

### Monitoring sensory

Sensory analysis, pH, total volatile basic nitrogen (TVB-N) and aerobic plate count are among several methods to detect spoilage. The onset of food spoilage was 2 overall sensory score (dislike), pH more than 6.4, TVB-N more than 20 mg/100 g (ES 1651/2005) and aerobic plate count more than log 7.0 cfu g^− 1^ (Zhang et al. 2016).

**Fig. 5:**
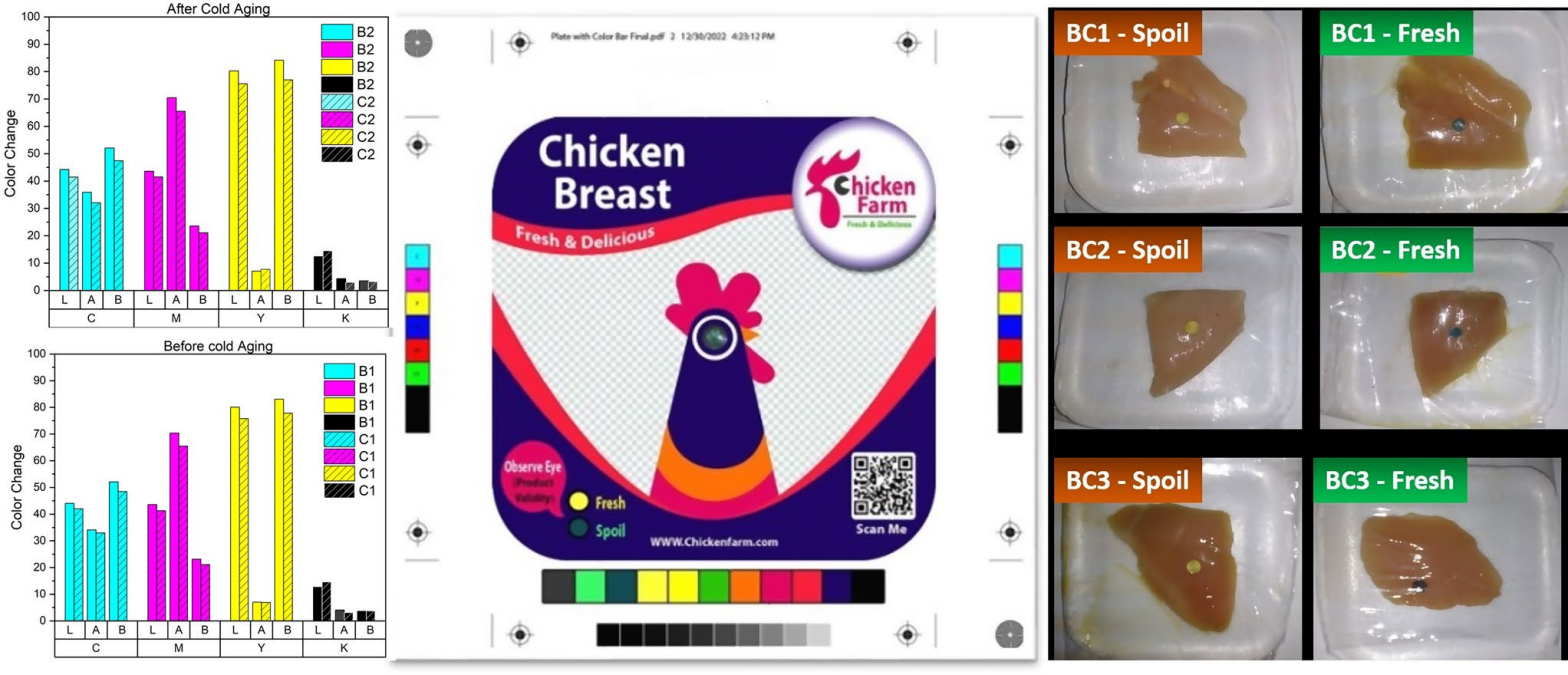
Chicken breast fillet storage samples with spoilage indicator in three different ratios BC1, BC2 and BC3 as fresh and after spoilage process and the design of packaging label

In the first trial, the indicator color changed when the pH of the chicken breast reached 7.1, TVB-N reached 79 mg/100 g and aerobic plate count reached log 7.78 cfu g^− 1^. These results indicated that the indicator color was changed far away when the spoilage processes started as shown in Table 2. The overall sensory score confirmed this recording score “very dislike” (1). Consequently, the indicator needed an improvement to detect the spoilage early after spoilage as seen in Fig. 5.

**Table 2:** Results (mean*± SD) of chicken breast at the time of color change of pH indicator

Sample	BC Mol/L	Overall acceptance	pH	TVN	APC
BC1	0.01	1	7.10 ± 0.0404	79.0 ± 2.0	7.78 ± 0.018
BC2	0.03	2	6.65 ± 0.015	70.0 ± 2.0	6.78 ± 0.03
BC3	0.05	2	6.55 ± 0.010	65.0 ± 1.803	6.45 ± 0.24

* Mean of 3 replicates

In the second and third trials, the indicator color changed in the package when the overall sensory score of the chicken breast was 2 “dislike”, the pH reached 6.65 and 6.55, and APC reached log 6.78 and log 6.45 cfu g^− 1^, respectively. There were significant (*P* < 0.5) correlations between changes in pH and APC (0.8) and between pH and sensory score (0.82), which indicate that pH can be relied upon for determining of beginning of spoilage. TVB-N was far from the maximum permissible limit as it reached 70 and 65 mg/100 g, but it is still much better than the first trial.

This color indicator of spoilage guarantees the consumer confidence through the removal of the spoiled food products as soon as the appearance of the signs of spoilage.

### Cool aging effect in printing label

The effect of cooling storage on the items during costumer marketing has a great importance. For that, color measurements were performed as the rate change of colors (∆E) for the packaging label before and after cooling storage for 15 days at 4 ° C. Four colors were measured with X-Rite, as shown in Fig. 5 and the results were tabulated in the Table 3.

**Table 3:** Rate change of colors after and before the cool aging

	C			M					Y				K	
	L	A	B	L		A		B	L	A	B	L	A	B
**B1**	44.04	-34.12	-52.08	43.57	70.33			-23.12	80.07	-7.02	83.09	12.58	-4.05	-3.62
**B2**	44.24	-35.88	-52.1	43.62	70.44			-23.6	80.25	-7.05	84.17	12.37	-4.33	-3.47
**∆E1**	∆ 0.68			∆ 0.18					∆ 0.26			∆ 0.39		
**C1**	42.01	-32.96	-48.47	41.25			65.48	-21.07	75.75	-6.91	77.84	14.59	-3.1	-3.7
**C2**	41.47	-32.04	-47.4	41.5			65.73	-21.54	75.53	-7.72	76.94	14.52	-2.98	-3.2
**∆E2**	∆ 0.61			∆ 0.35					∆ 0.56			∆ 0.44		
**∆E3**	∆ 3.03			∆ 2.29					∆ 3.75			∆ 2.13		
**∆E4**	∆ 2.13			∆ 2.45					∆ 3.26			∆ 1.85		

B1: The sample is without a polyester layer, B2: The sample without a polyester layer in the refrigerator, C1: The sample is covered with a polyester film, C2: The sample is covered with a polyester layer inside the refrigerator, ∆E1: Color change rate, between the samples, B1, B2, ∆E2: Color change rate, between the samples, C1, C2, ∆E3 Color change rate, between the samples, C2, B2, ∆E4 Color change rate, between the samples, C1, B1

According to the calculation and measurement, the differences in ∆E1 between sample B1 and B2 (∆ 0.18– ∆ 0.68) for printed color before and after cool storage was 0.5. Where, the variances in ∆E2 between sample C1 and C2 (∆ 0.35– ∆ 0.61) that indicated to tiny change in color appearance.

In addition, the differences in ∆E3 between the samples, C2, B2 (∆ 2.13– ∆ 3.75) for cool storage in the refrigerator was not affected on the print quality. ∆E4 between the samples, C1, B1 (∆ 1.85– ∆ 3.26) coated with the polyester layer was not change color quality. Coated with a polyester layer affected relatively the color quality, but within the limits of the specification. The samples covered with the polyester layer inside and outside the refrigerator of packaging label were not changed through cool storage as costumer demand. All measurements are within the permissible limits according to ISO 12647-6. This objective was achieved by covering with a polyester film to protect the color of the print area from handling and friction, as well as storage in refrigerators.

## Conclusion

Bromothymol was successfully encapsulated in nano-size with perfect unimodal particle size distribution. Three concentration of bromothymol indicator was applied to optimize the best sensory bromothymol capsules BC labels. Intelligent PE packaging with smart label as a flat responsive color eye relative to pH change was visually detected. The pH region of the color change could be explained using UV-Vis maximum absorption shift. POM studied texture of EBT films confirmed a homogenized distribution overall the film surface. The microbial activity to spoil chicken fillet was detected according to bacterial growth and pH changes with range from 70 to 65 mg/100 g. Overall previous investigations, EBT labels is successfully satisfied the diagnostic and smart response for food quality in chilled chicken lean. Thus, commercially production for EBT labels is recommended. The applied methodology used during this research article could be generalized to other indicator pH dye modules with highly promising results for future designed of pH sensitive dye applications as diagnostic smart color change food packaging materials. Nanocapsules have great emphasizes in formation the polymer films to be used as smart pad monitors and biosensors. Word health organization (WHO) and food & drug administration (FDA) should state a regulation of using an intelligent diagnostic label in all food packaging materials as compulsory action.

## Data Availability

Not applicable.
